# How does rising housing price affect the health of middle-aged and elderly people? The complementary mediators of social status seeking and competitive saving motive

**DOI:** 10.1371/journal.pone.0243982

**Published:** 2020-12-15

**Authors:** Wei Yuan, Shuying Gong, Yimin Han

**Affiliations:** 1 School of Business Administration, Hubei University of Economics, Wuhan, China; 2 College of Business, Shanghai University of Finance and Economics, Shanghai, China; University of Western Australia, AUSTRALIA

## Abstract

Under the backdrop of China’s aging population and continuous rising housing price and base on theories pertaining to social status seeking, marriage matching and intergenerational family relationships, use the 2010 and 2014 CFPS national survey micro data, we examine the impact of rising housing price on the health of middle-aged and elderly people and the underlying mechanisms. Rising housing price has a significant negative impact on the health of middle-aged and elderly people, and this effect is also reflected in their physical health, mental acuity and emotional well-being. The internal mechanism is that social status seeking motivation plays a significant mediator role. Through further analysis, we find that competitive saving motive is another intermediate mechanism that causes rising housing price to affect the health of middle-aged and elderly people; it is complementary to the social status seeking motivation. What’s more, the mediation effect of the competitive saving motive is notably heterogeneous, as it exists only for middle-aged and elderly people with male or noncollege educated child but does not exist for those with female or college educated child.

## Introduction

Individual health has been a research hotspot worldwide during the past decade. In 2009, the United Nations Development Program (UNDP) included “Health and Longevity” in the Human Development Index [1]. Since then, the volume of health literatures has grown immensely, and previous scholars have expanded their literature reviews [2,3]. Most researchers explore the factors affecting individuals’ health, these researches can be roughly divided into two categories. First, many studies have focused on the influence of macro factors (e.g., GDP, income gap, urbanization, economic and political regime, health insurance, education investment, and taxation) on individuals’ health [4–10]. This category represents the research mainstream, and the relationship between income gap and health inequality is the most popular topic. Some have proposed that the income gap harms individuals’ health [11], while some have found the opposite, that the income gap benefits individuals’ health [12], and others find no significant relationship between them [13]. Second, existing literatures have explored whether personal characteristics (age, gender, education level, marital status, etc.), lifestyle (smoking, exercise, etc.), and family characteristics (whether the household uses tap water, type of fuel used, etc.) influence individuals’ health from a microscopic perspective [14].

Although scholars have studied individuals’ health in depth from different perspectives with far-reaching conclusions, the existing literatures have research gaps.

First, as early as 1946, the World Health Organization indicated that health involves not only physical health aspect but also emotional well-being aspect [15]. Recently, the Guanghua Post-Consumer Big Data Center announced its health report, which indicated that the number of worldwide patients with dementia is over 24 million, increasing (on average) every 7 seconds. China’s outlook is also pessimistic in this regard. The data shows that Alzheimer’s disease in China accounts for about one-quarter of the world’s total cases, with an average of 300,000 new cases each year. Because dementia patients have damaged brains, base on this report as well as the actual situation, we propose that besides physical health and emotional well-being, mental acuity should be included in a complete assessment of individual health. However, many scholars, especially economic scholars, focus only on exploring the physical health of individuals, but pay little attention to their mental acuity and emotional well-being.

Second, although some scholars have studied the health of the elderly [16,17], few have focused on the health of middle-aged people. The reasons may be as follows: middle-aged people fall between the youth and the elderly; as a result, this group is often neglected. However, the middle-aged group will transition into the elderly. As China’s aging degree deepens, and since the upbringing and supporting mode of Chinese family, most of the middle-aged people with a child facing some new pressures, that is need to make material preparations for kinds of expenditures in advance (such as child’s education, medical care and transportation, etc.), so it is important to consider the physical health, mental acuity and emotional well-being of the middle-aged group during this transition period. Therefore, in terms of research objects, we propose the necessity of dividing the study population into middle-aged and elderly groups.

Third, according to data from the China Real Estate Statistical Yearbook, from 1991 to 2017, the average price of residential commercial housing in China rose from 786 RMB per square meter to 7,203 RMB, an increase of 916.41%. China’s continuous rising housing price [18] has become a major problem in China’s macro economy [19]. Some scholars have found that rising housing price has many negative effects, such as inhibiting consumption, hindering entrepreneurship, expanding income gaps and crowding out risky asset investments [18,20,21], and some scholars have subdivided the impact into “wealth effects”, “credit effects”, “alternative effects”, and "house slave effects” [19,21]. Currently, rising housing price is a typical phenomenon in China, but few scholars have linked it to individual health or explored the relationship between them.

Based on the existing framework, we focus on middle-aged and elderly people. First, we explore the relationship between rising housing price and the health of middle-aged and elderly people. To comprehensively analyze the effect of rising housing price on the health of middle-aged and elderly people, we innovatively categorize the health into three parts: physical health, mental acuity and emotional well-being. In addition, we aim to reveal the internal psychological mechanism between rising housing price and the health of middle-aged and elderly people. Finally, from the perspectives of marriage matching and intergenerational family relationships, we further analyze impact mechanisms and conduct a heterogeneity analysis to identify whether rising housing price affects the health of middle-aged and elderly people through other intermediate mechanisms, and evaluate the different influences between groups.

Through a series of theoretical and empirical analyses, the results show the following: First, rising housing price has a significant negative impact on the health of middle-aged and elderly people. This effect is not only reflected in their physical health but also in their mental acuity and emotional well-being. The internal mechanism is that social status seeking motivation has a significant mediation effect. Additionally, rising housing price has a significant “bidding effect” on the health of middle-aged and elderly people. Specifically, rising housing price harms the health of middle-aged and elderly people by motivating them to save competitively. However, the bidding effect only exists for middle-aged and elderly people with a male or noncollege educated child; it does not exist for middle-aged and elderly people with a female or college educated child.

Our paper makes the following contributions: First, we confirm that rising housing price has a significant negative impact on the health of middle-aged and elderly people, which is the first to analyze the relationship between rising housing price and the health of middle-aged and elderly people. This is pertinent given China’s aging population and rising housing price, and expands the scope of health impact factor researches, sets the stage for the future exploration of rising housing price as it relates to health inequality. Second, based on the social status seeking theory, we find that the motivation of seeking social status has a significant mediation effect for rising housing price on the health of middle-aged and elderly people. From one perspective, this finding helps the public and scholars understand the relationship between rising housing price and the health of middle-aged and elderly people. Additionally, the finding supports evidence of “Face Awareness (Consciousness)” of Chinese individuals from a micro perspective. Third, based on previous studies, this paper uses an indirect method to find that both social status seeking and competitive saving motive are intermediate factors that cause rising housing price to affect the health of middle-aged and elderly people. These findings help enrich theories involving marriage matching and intergenerational family relationships, as well as give new evidences for the competitive saving motive theory proposed by Wei and Zhang [22] based on the marriage market. Fourth, based on heterogeneity analysis, we find that the mediation effect of the competitive saving motive is significantly different with the child’s gender and educational level for middle-aged and elderly people. Specifically, the mediation effect only exists for middle-aged and elderly people with a male or noncollege educated child, but it does not exist for middle-aged and elderly people with a female or college educated child. These results reveal that, under the same conditions, males and females in the Chinese marriage market have significantly different bargaining power, and educational level can compensate some shortcomings in the marriage matching process. Fifth, we divide health into physical health, mental acuity and emotional well-being. This gives mental acuity and emotional well-being some due attention, to better describe individuals’ current health status; and it also has implications for future scholars to develop health definitions for these categories.

## Theory and hypotheses

Although scholars have not directly considered the relationship between rising housing price and individuals’ health, researches and related theories proposed by scholars help us identify their relationship. Specifically, rising housing price significantly influences individual housing assets, which inevitably differentiate individual incomes [18]. Since middle-aged and elderly people are the main homeowners in China, the rising housing price differentiates the income gap of this group by affecting their housing assets. Studies have confirmed that a widening income gap is negatively correlated with health [9,10]. Rising housing price may eventually harm the health of middle-aged and elderly people. As such, we present the first hypothesis:

**Hypothesis 1: Rising housing price has a significant negative impact on the health of middle-aged and elderly people**.

Housing plays a pivotal role in China. Wu et al. [23] pointed out that housing is the most important assets for urban residents in China. Housing makes up approximately 90% of the total assets of Chinese urban households [24]. Currently, besides the residence function, housing brings not only physical resources (e.g., child’s education, medical care and transportation) but also emotional benefits such as “a sense of belonging” and “security”, that elevate its importance. Today, housing has become one of the most important factors in residents’ social status [23]. To some extent, housing ownership has become one of the most important indicators for measuring an individual’s social status.

Social status mainly refers to the rank of an individual (or group) according to the degree of social identity [25]; Status seeking is defined as an individual or group’s pursuit of higher social status [26]. The former is a basic human need [27], and the latter is a basic human motivation [28]. Social status seeking theory postulates that people seek higher social status not only to satisfy psychological needs, such as improving self-esteem and efficacy [29] but also to obtain higher social status and obtain more resources (to further form the “club effect”) [30]. Zhang et al. [18] pointed out that rising housing price leads to an increased income gap, which significantly increases the motivation for achieving social status seeking [26]. Therefore, we further propose that rising housing price increases the income gap, and thus strengthens the social status seeking motivation of individuals. As discussed above, rising housing price has differentiated the incomes gap of middle-aged and elderly people. Based on this, we further propose that rising housing price broadens the income gap between middle-aged and elderly people, and then promotes their social status seeking motivation. In short, rising housing price helps strengthen the social status seeking motivation of middle-aged and elderly people. Since owning a house is an effective way to improve social status [22], under the strong social status seeking motivation, middle-aged and elderly people tend to accumulate more wealth by reducing necessary expenses for daily life and holding more than one job, to afford the housing price gap caused by rising housing price. However, these behaviors may eventually result in impaired health of middle-aged and elderly people. In summary, rising housing price strengthens the social status seeking motivation of middle-aged and elderly people, but this motivation may harm their health. This shows that rising housing price can influence the health of middle-aged and elderly people through social status seeking motivation. Based on this, we propose the second hypothesis:

**Hypothesis 2: Social status seeking motivation may play a significant mediator role in the health of middle-aged and elderly people affected by rising housing price**.

## Data, variables, and model

### Data

We use the data from the China Family Panel Studies (CFPS), a micro database organized by the China Social Science Research Center of Peking University. The samples are distributed in 25 provinces (municipalities and autonomous regions) in China, with the goal of tracking community, family and individual economic information. In turn, the study reveals dynamic trends in China’s economy, society, health and education. The micro database contains a total of four phases (2010, 2012, 2014, 2016) of national survey data, and we mainly use the 2010 and 2014 data for empirical analysis. The main reasons for using data from these two phases are as follows: first, we divide the health of middle-aged and elderly people into three dimensions: physical health, mental acuity and emotional well-being, and only the 2010 and 2014 data contain the necessary measurement items. Second, we need to use community variables in different research sections, and only the 2010 and 2014 data contain community information.

To explore our research questions, based on prior studies [10,31] we sort the observations as follows: (1) match and merge 2010 and 2014 CFPS databases (contain community, family, and adult); (2) delete observations under 40 years old in the combined data^①^, and retain observations of middle-aged and elderly people; (3) delete observations with missing important variables; (4) the important variables are adjusted to a significance level of 1% *Winsorize* to avoid abnormal values that may affect the results. In the end, 34,000 observations are obtained.

### Variables

#### Dependent variable

The health status of middle-aged and elderly people is the dependent variable. Prior scholars in economics and sociology have studied the health status (mainly physical health) of individuals; however, we also consider an individual’s mental acuity and emotional well-being. We divide the health of middle-aged and elderly people into three dimensions: physical health, mental acuity and emotional well-being. We use a method widely used in academic when defining physical health, that is, using individual self-assessment health to measure. Specifically, to define physical health, the samples answer the question, “What do you think of your health?” The answers “quite healthy”, “very healthy” and “healthy” are classified as “high physical health level” and assigned the value of 1; while “normal” and “unhealthy” are classified as “low physical health” and assigned the value of 0. To define mental acuity, refer to Zhou et al. [9] the samples answer the question “Can you remember the main things that happened to you in a recent week?” The answers, “remember all” and “can remember most” are classified as “high mental acuity level” and assigned the value of 1; while “can remember half”, “can only remember a few” and “can only remember a little bit” are classified as “low mental acuity level”, and assigned the value of 0 [9]. To define emotional well-being, we use the average value from the samples’ corresponding answers to the following two questions: “How often have you felt upset, depressed, and unable to do anything in the last month?”, and “How often have you felt nervous in the last month?” The values of the answers to these two questions are set to 1 for “almost every day”, 2 for “often”, 3 for “one-half of the time”, 4 for “some of the time”, and 5 for “never”. In addition, to ensure the accuracy of the results, we use various methods to define the different health dimensions.

#### Independent variable

Prior studies have used the average price of provincial residential commercial housing to represent housing price changes [23,32,33]. Considering the relativity and dynamics of rising housing price, which leads to continually increasing expectations [23], using the average price of residential commercial housing may not fully capture the dynamics. In view of this, based on prior studies, we use the natural logarithm of the average annual growth rate of residential commercial housing price over the past five years to measure rising housing price. Similarly, to avoid the influence of variable measurement bias on the results accuracy, we also use the average annual growth rate of residential commercial housing price over the past five years, housing price-income ratio and housing price index to measure rising housing price in the robustness test.

#### Mediator variable

Social status seeking motivation is the mediator variable. Various existing studies use different methods to define social status seeking motivation. Most of the studies use relationship expenditure to measure the social status seeking motivation [9,26,34,35]. While from one perspective, relationship expenditure is usually closely related to local customs and individual social connections, it shows reference group effects and regional effects in China. From the other perspective, relationship expenditure may be individual involuntary expenditure in some cases, while social status seeking motivation is an important manifestation of individual self-consciousness, and it shows initiative. Therefore, using relationship expenditure to represent social status seeking motivation may lead to potential endogenous problems due to measurement bias.

Aspiration Level Theory suggests that individuals’ material cravings can be reflected by comparing actual income status with self-reported income status [36,37]. According to this opinion, we believe that comparing actual social status with self-evaluated social status can be used to measure individual’s social status seeking motivation. Based on the material craving method definition by Zhou et al. [38], we use a more appropriate method than relationship expenditure to define social status seeking motivation. The specific method is as follows. First, based on the samples’ answers “What is your social status in the local area?” We assign a value of 1 for the response of “very low”, 2 for the response of “low”, 3 for the response of “general”, 4 for the response of “high”, and 5 for the response of “very high”, to obtain the self-evaluated social status. Second, based on the CFPS data obtained from the screening, with district as the unit, we divide individual income into 10 groups and rank them from low to high. We use 1–10 to indicate the actual social status of the samples in the local area (district)^②^; 1 represents “the lowest actual social status in the local area”, and 10 represents “the highest actual social status in the local area”. Finally, we use the ratio of actual social status to self-evaluated status obtained above to measure social status seeking motivation. The larger the ratio, the higher the difference between self-evaluated social status and actual social status, indicating that the individual’s social status seeking motivation is more intense.

#### Control variables

According to previous studies, the following control variables may affect the health of middle-aged and elderly people: individual-level factors, which include gender, age, years of education, smoking, exercise; family-level factors, which include income and housing ownership; and city-level factors, including whether it is a municipality or an eastern region.

Table 1 presents descriptive statistics for all variables. According to Table 1, the average physical health, mental acuity and emotional well-being of middle-aged and elderly people in China are 0.856, 0.751 and 4.328 respectively, indicating that most middle-aged and elderly people in China are in good health. To observe the relationship between rising housing price and the health status of middle-aged and elderly people, we use Stata15.0 to draw scatter plots (due to length limitations, scatter plots are not part of this manuscript, but are available from the authors). These figures show that there is a significant negative correlation between rising housing price and physical health, mental acuity and emotional well-being of middle-aged and elderly people. To further verify these relationships, we will conduct a more in-depth analysis next.

**Table 1:** Descriptive statistics.

Variable	Observation	Mean	S.D.	Min	Max
Dependent Variables					
Physical Health	86580	0.856	0.351	0	1
Mental Acuity	86580	0.751	0.432	0	1
Emotional Well-Being	41431	4.328	0.841	1	5
Independent Variable					
Rising Housing Price	86580	3.122	0.640	0.693	4.001
Mediator Variable					
Social Status Seeking Motivation	39446	2.751	2.115	0.2	10
Control Variables: Individual Level					
Gender (Male = 1)	44085	0.493	0.500	0	1
Age	44066	56.138	11.411	40	110
Years of Education	59230	5.189	4.848	0	22
Marital Status (Married = 1)	39019	0.982	0.132	0	1
Non-agricultural Population (Yes = 1)	43.586	0.289	0.453	0	1
Smoking (Yes = 1)	86580	0.330	0.470	0	1
Exercise (Yes = 1)	86580	0.668	0.471	0	1
Endowment Insurance (Yes = 1)	44085	0.337	0.477	0	1
Medicare (Yes = 1)	43650	0.883	0.322	0	1
Control Variables: Family Level					
Log of Per Capita Income	73337	8.611	1.478	0.405	11.124
Housing Ownership (Yes = 1)	86580	0.955	0.207	0	1
Urban Population (Yes = 1)	85493	0.434	0.496	0	1
Whether the Household Uses Tap Water (Yes = 1)	78330	0.594	0.491	0	1
Type of Fuel Used	78331	2.673	1.757	1	7
Household Power Used	78323	3.505	0.587	1	4
Control Variables: Child Level					
Child’s Gender (Male = 1)	37346	0.615	0.487	0	1
Child’s Age	37173	29.334	11.034	0	77
Child’s Years of Education	36062	8.676	4.248	0	22
Child’s Marital Status (Married = 1)	37353	0.562	0.496	0	1
Control Variables: City Level					
Municipality (Yes = 1)	86580	0.093	0.29	0	1
Eastern area (Yes = 1)	86580	0.413	0.492	0	1

### Model

#### The main effect model

To verify the impact of rising housing price on the health status (physical health, mental acuity and emotional well-being) of middle-aged and elderly people, we construct the following regression models:

$$P_{r}({Hphysiological}_{i}=1)=G(\beta_{0}+\beta_{1}{Hp}_{i}+\Gamma X_{i})$$ (1)

$$P_{r}({Hmental}_{i}=1)=G(\beta_{0}+\beta_{1}{Hp}_{i}+\Gamma X_{i})$$ (2)

$${Hspirit}_{i}=\beta_{0}+\beta_{1}{Hp}_{i}+\Gamma X_{i}+\varepsilon_{i}$$ (3)

Among them, model (1) and model (2) are mixed *Probit* models, and model (3) is a *Pols* model. The dependent variable *Hphysiological*_*i*_ in model (1) indicates the physical health status of individual *i*, the dependent variable *Hmental*_*i*_ in model (2) indicates the mental acuity status of individual *i*, and the dependent variable *Hspirit*_*i*_ in model (3) indicates the emotional well-being of individual *i*. The core independent variable in each model is *Hp*_*i*_, which represents the rising housing price in the local area of individual *i*. The coefficient *beta*_*1*_ in each model captures the effects of rising housing price on physical health, mental acuity and emotional well-being of middle-aged and elderly people, while *X*_*i*_ represents a series of control variables that may affect the health of middle-aged and elderly people.

#### Mediator effect model

We use the Causal Step Regression method proposed in Baron and Kenny [39] to test the mediation effect. First, we use the above construction model to test the effect of rising housing price on the health status of middle-aged and elderly people. Then, we use model (4) to perform a regression analysis between rising housing price and social status seeking motivation. Finally, we use model (5), model (6), and model (7) to examine the relationship between rising housing price, social status seeking motivation and the health status of middle-aged and elderly people.

$$M_{i}=\beta_{0}+\beta_{1}{Hp}_{i}+{\Gamma X}_{i}+\varepsilon_{i}$$ (4)

$$P_{r}({Hphysiological}_{i}=1)=G(\beta_{0}+\beta_{1}{Hp}_{i}+\beta_{2}M_{i}+\Gamma X_{i})$$ (5)

$$P_{r}({Hmental}_{i}=1)=G(\beta_{0}+\beta_{1}{Hp}_{i}+\beta_{2}M_{i}+\Gamma X_{i})$$ (6)

$${Hspirit}_{i}=\beta_{0}+\beta_{1}{Hp}_{i}+\beta_{2}M_{i}+\Gamma X_{i}+\varepsilon_{i}$$ (7)

Among them, *M*_*i*_ indicates the social status seeking motivation of individual *i*, and the other variables are set as above. To further validate the results of the mediation effect, we also use the “*Bootstrap*” method, which is currently widely accepted and used to test mediation effects [40,41].

## Empirical results

### Results of main effect

Table 2 shows how rising housing price affects the health of middle-aged and elderly people^③^. The results suggest that rising housing price has a significant negative impact, not only on the physical health of middle-aged and elderly people (*beta* = -0.575, *p<*0.01) but also on their mental acuity (*beta* = -0.198, *p<*0.01) and their emotional well-being (*beta* = -0.092, *p<*0.01). Specifically, a 1% increase in housing price leads to a 16.92% increase in the probability of physical health of middle-aged and elderly people moving into the 0 category (“general” and “unhealthy”), and a 7.31% increase in the probability of mental acuity of the middle-aged and elderly people moving into the 0 category (“can remember half”, “only remember a few” and “can only remember a little”), and a significantly decrease of 0.092 unit in emotional well-being. The above results show that rising housing price has a significant negative impact on the health of middle-aged and elderly people, and this effect is reflected in their physical health, mental acuity and emotional well-being. As a result, hypothesis 1 is supported.

**Table 2:** The effects of rising housing price on the health of middle-aged and elderly people.

Dependent Variable	Physical Health		Mental Acuity		Emotional Well-Being	
Model	*probit*		*probit*		*pols*	
Column	(1)		(2)		(3)	
Rising Housing Price	-0.575***	(0.014)	-0.198***	(0.012)	-0.092***	(0.007)
Age	-0.020***	(0.001)	-0.011***	(0.001)	0.002***	(0.001)
Gender	0.213***	(0.019)	0.063***	(0.018)	0.140***	(0.012)
Years of Education	0.030***	(0.002)	0.045***	(0.002)	0.011***	(0.001)
Marital Status	0.303***	(0.055)	0.098*	(0.054)	0.172***	(0.035)
Non-agricultural Population	0.010***	(0.021)	0.156***	(0.019)	0.015	(0.012)
Smoking	-0.036*	(0.020)	-0.208***	(0.018)	0.016	(0.012)
Exercise	0.022	(0.017)	0.197***	(0.016)	0.036***	(0.010)
Insurance	-0.061***	(0.017)	-0.047***	(0.016)	0.001	(0.010)
Medicare	-0.041	(0.025)	0.078***	(0.022)	0.067***	(0.014)
Per Capita Income	0.026***	(0.006)	0.056***	(0.005)	0.030***	(0.003)
Housing Ownership	-0.006	(0.041)	0.056	(0.035)	0.049**	(0.022)
_Cons	2.807***	(0.096)	0.231***	(0.087)	3.776***	(0.056)
Province Dummy	Yes		Yes		Yes	
*R*^2^	0.095		0.068		0.031	
Observation	34442		34442		33147	

Notes: 1. The results reported in column (1) and column (2) are only the original estimation coefficient, not the marginal effect. 2. The value in parentheses is the standard error. 3.***, **, * represent 1%, 5%, and 10% significance levels.

### Mediation effect results

Table 3 presents the results of the mediation effect by using the Causal Step Regression method. The results show that rising housing price in column (1) significantly negative affects the physical health of middle-aged and elderly people (*beta* = -0.575, *p*<0.01), indicating that rising housing price has a direct negative impact on the physical health of middle-aged and elderly people. Column (2) suggests that rising housing price significantly positive affects social status seeking motivation (*beta* = 0.054, *p*<0.01), which reflects that social status seeking motivation of middle-aged and elderly people increases as housing price increases. Column (3) shows that rising housing price significantly negative affects physical health (*beta* = -0.566, *p*<0.01). Additionally, the result shows that social status seeking motivation significantly negative affects the physical health of middle-aged and elderly people (*beta* = -0.038, *p*<0.01). In summary, according to the results of columns (1) to (3), the mediation effect of social status seeking motivation is significant, and it plays a partial mediator role in the impact of rising housing price on physical health of middle-aged and elderly people. Similarity, according to the results of columns (4) to (6), the mediation effect of social status seeking motivation is significant, and it plays a partial mediator role in the impact of rising housing price on mental acuity of middle-aged and elderly people; according to the results of columns (7) to (9), the mediation effect of social status seeking motivation is significant, and it plays a partial mediator role in the impact of rising housing price on emotional well-being of middle-aged and elderly people. All in all, the research results obtained above confirm that the mediation effect of social status seeking motivation is significant, that is, rising housing price can indirectly affect the health status of middle-aged and elderly people through it. These results also show that rising housing price can also affect the health of middle-aged and elderly people through other intermediary variables. According to the principle of the mediation effect proposed by Zhao et al. [40], since the coefficients of rising housing price in columns (2), (5) and (8) are positive, and the coefficients of rising housing price and social status seeking motivation are negative in columns (3), (6) and (9), the one positive coefficient is multiplied by two negative coefficients, resulting in a positive value, indicating that social status seeking motivation has other complementary mediator^④^. To further reveal the potential mechanism of rising housing price affecting the health of middle-aged and elderly people, we consider the perspective of marriage matching competition theory and intergenerational family relationships, under the backdrop of Chinese marriage market culture.

**Table 3:** The mediation effect results based on causal step regression analysis.

Dependent Variable	Physical Health	Social Status Seeking Motivation	Physical Health	Mental Acuity	Social Status Seeking Motivation	Mental Acuity	Emotional Well-Being	Social Status Seeking Motivation	Emotional Well-Being
Path	X--Y	X--M	X,M--Y	X--Y	X--M	X,M--Y	X--Y	X--M	X,M--Y
Model	*probit*	*pols*	*probit*	*probit*	*pols*	*probit*	*pols*	*pols*	*pols*
Column	(1)	(2)	(3)	(4)	(5)	(6)	(7)	(8)	(9)
Rising Housing Price	-0.575***	0.054***	-0.566***	-0.198***	0.054***	-0.234***	-0.092***	0.054***	-0.089***
	(0.014)	(0.009)	(0.014)	(0.012)	(0.009)	(0.012)	(0.007)	(0.009)	(0.007)
Social Status Seeking Motivation			-0.038***			-0.043***			-0.042***
			(0.008)			(0.007)			(0.005)
Age	-0.020***	-0.009***	-0.020***	-0.011***	-0.009***	-0.012***	0.002***	-0.009***	0.002***
	(0.001)	(0.001)	(0.001)	(0.001)	(0.001)	(0.001)	(0.001)	(0.001)	(0.001)
Gender	0.213***	0.259***	0.195***	0.063***	0.259***	0.182***	0.140***	0.259***	0.152***
	(0.019)	(0.014)	(0.020)	(0.018)	(0.014)	(0.018)	(0.012)	(0.014)	(0.012)
Years of Education	0.030***	0.006***	0.030***	0.045***	0.006***	0.050***	0.011***	0.006***	0.011***
	(0.002)	(0.001)	(0.002)	(0.002)	(0.001)	(0.002)	(0.001)	(0.001)	(0.001)
Marital Status	0.303***	-0.117***	0.299***	0.098*	-0.117***	0.168***	0.172***	-0.117***	0.169***
	(0.055)	(0.042)	(0.058)	(0.054)	(0.042)	(0.056)	(0.035)	(0.042)	(0.035)
Non-agricultural Population	0.010***	0.355***	0.093***	0.156***	0.355***	0.228***	0.015	0.355***	0.029**
	(0.021)	(0.014)	(0.021)	(0.019)	(0.014)	(0.019)	(0.012)	(0.014)	(0.012)
Smoking	-0.036*	-0.045***	-0.093***	-0.208***	-0.045***	-0.011	0.016	-0.045***	0.015
	(0.020)	(0.014)	(0.021)	(0.018)	(0.014)	(0.019)	(0.012)	(0.014)	(0.012)
Exercise	0.022	-0.081***	0.062***	0.197***	-0.081***	0.052***	0.036***	-0.081***	0.033***
	(0.017)	(0.012)	(0.018)	(0.016)	(0.012)	(0.017)	(0.010)	(0.012)	(0.010)
Insurance	-0.061***	-0.102***	-0.076***	-0.047***	-0.102***	-0.058***	0.001	-0.102***	-0.004
	(0.017)	(0.012)	(0.017)	(0.016)	(0.012)	(0.016)	(0.010)	(0.012)	(0.010)
Medicare	-0.041	-0.079***	-0.042	0.078***	-0.079***	0.066***	0.067***	-0.079***	0.065***
	(0.025)	(0.017)	(0.026)	(0.022)	(0.017)	(0.023)	(0.014)	(0.017)	(0.014)
Per Capita Income	0.026***	0.030***	0.028***	0.056***	0.030***	0.056***	0.030***	0.030***	0.031***
	(0.006)	(0.004)	(0.006)	(0.005)	(0.004)	(0.005)	(0.003)	(0.004)	(0.003)
Housing Ownership	-0.006	-0.182***	-0.012	0.056	-0.182***	0.029	0.049**	-0.182***	0.040*
	(0.041)	(0.026)	(0.041)	(0.035)	(0.026)	(0.035)	(0.022)	(0.026)	(0.022)
_Cons	2.807***	1.474***	2.861***	0.231***	1.474***	0.230***	3.776***	1.474***	3.831***
	(0.096)	(0.067)	(0.099)	(0.087)	(0.067)	(0.091)	(0.056)	(0.067)	(0.057)
Province Dummy	YES	YES	YES	YES	YES	YES	YES	YES	YES
R^2^	0.095	0.073	0.093	0.068	0.073	0.075	0.031	0.073	0.034
Observation	34442	33147	33147	34442	33147	33147	33147	33147	32990

Notes: 1. The results reported in columns (1) and (2) are only the original estimation coefficient, not the marginal effect. 2. The value in parentheses is the standard error. 3.***, **, * represent 1%, 5%, and 10% significance levels, respectively. 4. The dependent variable in columns (2), (5), and (8) is social status seeking motivation.

In addition, to ensure the mediation effect results’ accuracy, we also refer to the mediation effect analysis program [40,41], use “*Bootstrap*” to develop the mediation effect robustness test. We select a sample size of 5000 in the “*Bootstrap*” method, and select Model 4 at 95% confidence. The results show that the partial mediation effect of social status seeking motivation exists in the relationship between rising housing price and (1) physical health (*LLCI* = 0.0002 *ULCI* = 0.0025), (2) mental acuity (*LLCI* = 0.0023 *ULCI* = 0.0051), and (3) emotional well-being (*LLCI* = 0.0016 *ULCI* = 0.0005) of middle-aged and elderly people. All three intervals do not contain 0, which also suggests a partial mediator. The results using the “*Bootstrap”* method are consistent with those obtained by the above-described Casual Step Regression method, which verify a robust mediation effect of social status seeking motivation on the relationship between rising housing price and the health of middle-aged and elderly people. Consequently, hypothesis 2 is supported.

### Robustness test

#### Endogenous test

The current study shows that rising housing price can significantly affect the health of middle-aged and elderly people through social status seeking motivation. Zhou et al. [9] pointed out that health can affect the income gap through human capital; and Li et al. [42] also pointed out that the income gap also significantly affects housing price, which indicates that the health of middle-aged and elderly people can also affect housing price through the human capital-income gap. There is a reverse causal relationship between rising housing price and the health of middle-aged and elderly people, which may lead to endogenous bias in the results. To avoid this potential endogenous problem, and ensure the results are accurate and reliable, we use the instrumental variable method to carry out the endogeneity.

Since rising housing price is often closely related to building costs, and “the daily wage of construction workers” is an important indicator of building costs. So, rising housing price is highly correlated with “the daily wage of construction workers”. And construction workers are part of a common occupational group in society, and the daily wage of this group may not directly affect the health of others, so “the daily wage of construction workers” should be irrelevant or have a low correlation with individual health. Therefore, “the daily wage of construction workers” can be an instrumental variable for rising housing price. To avoid outliers affecting the results, “the daily wage of construction workers” is adjusted to 1%*Winsorize*, and taken the natural logarithm. Additionally, we also use “the distance from the residence to the county (urban) center” and “the time spend from residence to the county (urban) center” as instrumental variables for rising housing price, and the process is the same as the daily wage of construction workers. To verify the feasibility of these three instrumental variables, we also conduct identification test and an exogenous test. The results show that “the daily wage of construction workers”, “the distance from the residence to the county (urban) center”, and “the time spend from residence to the county (urban) center” are effective and powerful instrumental variables.

#### Measurement change for the important variables

To exclude a potential measurement problem that affects the results, we also change the measurement of the core independent and dependent variables to conduct the robustness test. To change the measurement of the core independent variable (rising housing price), we use the “annual residential commercial housing price growth rate over the past five years” ^⑤^, “housing price-income ratio” and “housing price index” to measure rising housing price. These three substitutes have traditionally been widely used to measure rising housing price. To change the measurement of the dependent variables (physical health, mental acuity, and emotional well-being), we use the following questions in the CFPS questionnaire to measure each dependent variables: (1) “How do you think your health compares with the previous year?” A value of 1 is assigned for the response of “better”, a value of 0 for the response of “not change”, a value of -1 for the response of “worse”^⑥^ to measure physical health; (2) we use the average value from the following 6 questions to measure emotional well-being: “In the last month, how often do you feel depressed and do not want to do anything?” A value of 1 is assigned for the response of “almost every day”, a value of 2 is assigned for the response of “frequently”, a value of 3 is assigned for the response of “one-half of the time”, a value of 4 is assigned for the response of “sometimes”, and a value of 5 is assigned for the response of “never”(same below). The other five questions are: “In the last month, how often have you felt nervous?”, “How often have you felt uncomfortable and had difficulty remaining calm in the last month?”, “What is the frequency you feel unhopeful about the future during the last month?”, “How often do you have difficulty doing anything in the last month?”, and “How often have you felt life is meaningless in the last month?”. Since no question in the CFPS questionnaire measure mental acuity, we do not conduct a robustness test for mental acuity here; however, the other robustness tests can provide strong evidences for the research on mental acuity.

#### Addition of control variables

From previous studies, we observe that the control variables in each model setting vary. To avoid missing important variables in the model design that may affect the research results, we add control variables to the study that may affect the health of middle-aged and elderly people [9,10], such as “whether the household uses tap water”, “type of fuel used”, and “household power used”.

#### Change in the sample age range

We limit the age to 40 years and older when screening samples. Currently, there is no consistent age range definition standard for middle-aged and elderly people. For example, Chen and Zhang [10] defined the age range of middle-aged and elderly people as 45 years old and above; Wu et al. [21] defined the middle age range as over 35 and below 60 years old, and over 60 as elderly. To avoid the problem of samples self-selection bias, we rescreen the samples base on different age ranges for middle-aged and elderly people; specifically, we do the robustness tests of people’s age at “45 years old and above”, at “35 years old and above”, and “between 45 and 65 years old”.

Based on the above robustness tests, we obtain the regression results shown in Table 4. The results consistently demonstrate: (1) Rising housing price has a significant negative impact on the health of middle-aged and elderly people. Specifically, rising housing price significantly reduces the physical health, mental acuity, and emotional well-being of middle-aged and elderly people. (2) The mediation effect of social status seeking motivation is significant, which means that rising housing price significantly negative affects the health of middle-aged and elderly people through social status seeking motivation. Because the results of the robustness tests are consistent with the empirical analysis above, the results of this study have strong reliability and robustness.

**Table 4:** Results of various robustness tests.

Dependent Variable	Physical Health	Social Status Seeking Motivation	Physical Health	Mental Acuity	Social Status Seeking Motivation	Mental Acuity	Emotional Well-Being	Social Status Seeking Motivation	Emotional Well-Being
Path	X--Y	X--M	X,M--Y	X--Y	X--M	X,M--Y	X--Y	X--M	X,M--Y
Column	(1)	(2)	(3)	(4)	(5)	(6)	(7)	(8)	(9)
1. Endogenous test: using “the daily wage of construction workers” as an instrumental variable for rising housing price.									
Model	*iv_probit*	*iv_pols*	*iv_probit*	*iv_probit*	*iv_pols*	*iv_probit*	*iv_pols*	*iv_pols*	*iv_pols*
Rising Housing Price	-0.803***	0.041***	-0.786***	-0.203***	0.041***	-0.304***	-0.092***	0.041***	-0.090***
	(0.024)	(0.015)	(0.024)	(0.021)	(0.015)	(0.022)	(0.014)	(0.015)	(0.014)
Social Status Seeking Motivation			-0.034***			-0.034***			-0.040***
			(0.011)			(0.010)			(0.006)
Province Dummy	YES	YES	YES	YES	YES	YES	YES	YES	YES
R^2^		0.053			0.053		0.028	0.053	0.03
*Wald Chi2*	352.16***		325.07***	348.2***		25.10***			
Observation	22278	21322	21322	22278	21322	21322	21317	21322	21200
2. The core independent variable measurement is changed to use the average annual growth rate of housing price in commercial housing over the past five years to measure rising housing price.									
Model	*probit*	*pols*	*probit*	*probit*	*pols*	*probit*	*pols*	*pols*	*pols*
Rising Housing Price	-0.524***	0.054***	-0.516***	-0.178***	0.054***	-0.208***	-0.089***	0.054***	-0.086***
	(0.016)	(0.009)	(0.014)	(0.011)	(0.009)	(0.012)	(0.007)	(0.009)	(0.007)
Social Status Seeking Motivation			-0.039***			-0.044***			-0.042***
			(0.008)			(0.007)			(0.005)
Province Dummy	YES	YES	YES	YES	YES	YES	YES	YES	YES
R^2^	0.089	0.073	0.088	0.067	0.073	0.073	0.031	0.073	0.033
Observation	34442	33147	33147	34442	33147	33147	33147	33147	32990
3. The method of measuring the dependent variable is changed.									
Model	*oprobit*	*pols*	*oprobit*				*pols*	*pols*	*pols*
Rising Housing Price	-0.072***	0.054***	-0.068***				-0.069***	0.054***	-0.067***
	(0.010)	(0.009)	(0.010)				(0.006)	(0.009)	(0.006)
Social Status Seeking Motivation			-0.045***						-0.045***
			(0.007)						(0.004)
Province Dummy	YES	YES	YES				YES	YES	YES
R^2^	0.015	0.073	0.015				0.052	0.073	0.056
Observation	33470	33147	33142				33029	33147	32887
4. More control variables are added.									
Model	*probit*	*pols*	*probit*	*probit*	*pols*	*probit*	*pols*	*pols*	*pols*
Rising Housing Price	-0.585***	0.051***	-0.574***	-0.205***	0.051***	-0.242***	-0.101***	0.051***	-0.099***
	(0.014)	(0.009)	(0.014)	(0.012)	(0.009)	(0.012)	(0.007)	(0.009)	(0.007)
Social Status Seeking Motivation			-0.038***			-0.044***			-0.043***
			(0.008)			(0.007)			(0.005)
Province Dummy	YES	YES	YES	YES	YES	YES	YES	YES	YES
R^2^	0.096	0.074	0.094	0.068	0.074	0.076	0.034	0.074	0.036
Observation	34438	33144	33144	34438	33144	33144	33144	33144	32987
5. The age range of the samples are modified to 45–65 years old.									
Model	*probit*	*pols*	*probit*	*probit*	*pols*	*probit*	*pols*	*pols*	*pols*
Rising Housing Price	-0.574***	0.032***	-0.567***	-0.231***	0.032***	-0.257***	-0.097***	0.032***	-0.095***
	(0.017)	(0.011)	(0.018)	(0.014)	(0.011)	(0.014)	(0.009)	(0.011)	(0.009)
Social Status Seeking Motivation			-0.027***			-0.046***			-0.044***
			(0.010)			(0.009)			(0.006)
Province Dummy	YES	YES	YES	YES	YES	YES	YES	YES	YES
R^2^	0.085	0.082	0.084	0.064	0.082	0.068	0.033	0.082	0.036
Observation	22345	21710	21710	22345	21710	21710	21710	21710	21620

Notes: 1. In the endogenous robustness tests, we also use “the distance from the residence to the county (urban) center” and the “time spent from residence to the county (urban) center” as the instrumental variables of rising housing price. 2. In the change of the core independent variable robustness test, we also use the housing price-income ratio and the housing price index to measure the rising housing price. 3. In the change of the age range of samples robustness, we also control the sample at “35 years old and above”, “45 years old and above”. Due to length limitations, we do not report a complete set of robustness test results, but these can be obtained from authors. 4. In addition to the *Pols* model in the table, the results obtained based on other models are only the original estimated coefficients, not the marginal effects. 5. The value in parentheses is the standard error. 6.***, **, * represent the 1%, 5%, and 10% significance levels, respectively. 7. The dependent variable in columns (2), (5), and (8) is social status seeking motivation.

## Further mechanism analysis and heterogeneity analysis

### Further mechanism analysis

Our study shows that rising housing price has a significant negative impact on the health of middle-aged and elderly people (including physical health, mental acuity, and emotional well-being), and the social status seeking motivation has a mediation effect. In addition, according to the mediation effect test procedure and principle proposed by Zhao et al. [40], we find that there are other mediators that complement with the social status seeking motivation in the impact of rising housing price on the health of middle-aged and elderly people. Consider most middle-aged and elderly people in China have children, and the intergenerational family relationships are particularly prominent between Chinese parents and their children. Chinese parents are mostly concerned about their children’s marriage prospects, and housing as a social status symbol can effectively improve the success rate of marriage matching [22]. Based on these two issues, we consider rising housing price may increase the difficulty of purchasing housing, and enhance the degree of marriage-matching competition. Since children’s marriage is a prominent issue for Chinese parents, rising housing price may affect the health of Chinese parents by narrowing the range of their children’s potential marriage matching. But what is the underlying mechanism? To solve this problem and to further understand the relationship between rising housing price and the health of middle-aged and elderly people, we take the perspective of marriage matching competition and intergenerational family relationships to expand the following in-depth mechanism analysis based on the background of Chinese traditional marriage culture.

In China, housing plays an important role in the public life, and it is a carrier for demonstrating social status. Additionally, housing has an important influence on individual marriage matching. According to statistics, 41% of males and 57% of females think that it is necessary to prepare a separate house for marriage; and 60% of males and 51% of females say that they or their friends have suffered a breakup from someone who cannot afford to buy a house for marriage; this phenomenon is particularly significant in Zhengzhou, Shanghai and Tianjin^⑦^. In addition, only 20% of Chinese mothers allow their daughter to marry a male without a house^⑧^. These statistics support the results in the literatures [23,43], that find housing ownership is necessary for marriage matching in China, and indirectly indicate that owning a house can increase people’s competitiveness in the marriage market [22].

With the rapid economic development, although individuals’ material living standards have greatly improved, buying a house for marriage still requires a large sum of money. Especially as housing price continuously rises, the purchase of marriage housing has become a major consumption of the whole family. From a realistic perspective, it is likely that the married couple will need to obtain additional financial support from their parents and other elders after they have spent all their savings.

Chinese culture is different from foreign culture. Specifically, intergenerational family relationships are an outstanding social phenomenon in China, reflect in the relationship between the previous generation and the next generation, and in the relationship between one or many generations [44,45]. The main reason is that saving is an important mean of Chinese financial security to support in the old age [46], and child is an effective substitute for life cycle saving [47]. In essence, Chinese intergenerational family relationships are an upbringing and supporting mode instead of the Western “relay mode” [48]. In this special mode, the most important thing for the family is the child’s marriage. Given that a house can enhance the child’s competitiveness in the marriage market, parents usually put forth much effort to help a child buy a house for marriage. Researchers have noted that today, the phenomenon of “purchase a marriage house”, “adding dowry” and “preparing bride price” for marriage have become a rigid requirement for parents [49]. To fulfill these requirements, parents tend to make material preparations in advance [44,45], such as saving more money by reducing consumption or entrepreneurship [22,31,44,45,50,51].

To summarize, owning a house is an effective way for people to improve their competitiveness in the marriage market relative to their competitors [22]. For success in marriage matching, Chinese parents tend to save money to help their children purchase housing for marriage. This reflects the “bidding effect” of Chinese parents’ housing purchase behavior [52]. Further, rising housing price makes it more difficult to own a house; as a result, rising housing price has substantially strengthened the “bidding effect” of buying houses. However, the stronger “bidding effect” incents Chinese parents to be more inclined to save money to help their children buy houses for marriage. The “bidding effect” caused by rising housing price further stimulates the competitive saving motive of Chinese parents. Since Chinese parents are mainly middle-aged and elderly, we suggest that rising housing price presents a stronger incentive for the middle-aged and elderly to save competitively. Driven by strong competitive saving motive to buy a marriage house for their children, middle-aged and elderly people tend to overwork to save money for their children, possibly damaging their physical health. What’s more, to save money for their children to buy marriage houses, middle-aged and elderly people may suffer a huge burden [31], which may cause mental stress, depression and insomnia, and work against their mental acuity and emotional well-being. As a result, a stronger competitive saving motive may work against the health of middle-aged and elderly people.

In summary, rising housing price increases the competitive saving motive, which harms the health of middle-aged and elderly people. Based on the viewpoint, we suggest that competitive saving motive may be another mediation mechanism that negatively affects the health of middle-aged and elderly people. To identify this potential mediation mechanism in the impact of rising housing price on the health of middle-aged and elderly people, we mainly use the indirect proof method^⑨^. Yu and Lian [31] postulated that a son’s marriage is a natural event, and a son’s marital status (married/unmarried) provides a quasi-natural experiment to test the “marriage effect” affecting family savings. In this paper, the child’s (including son and daughter)^⑩^ marriage is regarded as a natural event, and the mediation effect of “competitive saving motive” is indirectly verified through the significant interaction between the child’s marital status and rising housing price. Specifically, we divide the samples into a “married group” (treatment group) and an “unmarried group” (control group) based on the child’s marital status, assigning married as 1 and unmarried as 0. According to Chinese traditional marriage culture, Chinese parents tend to save money to purchase a marriage house influenced by competitive saving motive to improve the marriage success rate of their children. Compared with Chinese parents with married children, the competitive saving motive of Chinese parents with unmarried children is much stronger. We have previously discussed that rising housing price strengthens competitive saving motive, which in turn impairs the health of middle-aged and elderly people. Therefore, under the circumstance that other conditions remain unchanged, if the competitive saving motive strengthened by rising housing price reduces the health of middle-aged and elderly people, as housing price rises, the health of middle-aged and elderly people with married child and those with unmarried child will be significantly different, the specific performance is that the former health is significantly better than the latter. In view of this, we attempt to identify whether the coefficient of interaction between rising housing price and child's marital status (1 = married, 0 = unmarried) is significant, and identify the mediation effect of competitive saving motive under the context of rising housing price, by examining the differences of significance between the health of middle-aged and elderly people with marriage and unmarried child.

Table 5 reports the results of the mediation effect of competitive saving motive. Column (1) shows that the interaction between rising housing price and child’s marital status^⑪^ (1 = married, 0 = unmarried) significantly positive affects the physical health of middle-aged and elderly people (*beta* = 0.061, *p*<0.05). Specifically, when the housing price rises by 1%, the physical health status probability of middle-aged and elderly people with unmarried children is (significantly) 1.9% worse than those with married child. Column (2) shows that the interaction between rising housing price and child’s marital status significantly positive affects the mental acuity of middle-aged and elderly people (*beta* = 0.041, *p*<0.05). Specifically, when housing price rises by 1%, the mental acuity probability of middle-aged and elderly people with unmarried children is (significantly) 1.5% worse than those with married child. Column (3) shows that the interaction between rising housing price and child’s marital status significantly positive affects the emotional well-being of middle-aged and elderly people (*beta* = 0.030, *p*<0.05). Specifically, when the housing price rises by 1%, the emotional well-being of middle-aged and elderly people with unmarried child is (significantly) worse by 0.03 unit than those with married children. The above results show that the negative impact of rising housing price on the health of middle-aged and elderly people with unmarried children is significantly higher than those with married child. The results indicate that the mediation effect of competitive saving motive is significant, and it is another mediation mechanism that affects the health of middle-aged and elderly people.

**Table 5:** The Mediation effect results of competitive saving motive.

Dependent Variable	Physical Health		Mental Acuity		Emotional Well-Being	
Model	*probit*		*probit*		*pols*	
Column	(1)		(2)		(3)	
Rising Housing Price	-0.383***	(0.017)	-0.121***	(0.016)	-0.045***	(0.007)
Interaction	0.061**	(0.026)	0.041*	(0.023)	0.030**	(0.015)
Child’s Age	0.002	(0.002)	-0.001	(0.002)	0.003**	(0.001)
Child’s Gender	0.005	(0.016)	0.005	(0.015)	-0.015	(0.010)
Child’s Years of Education	0.013***	(0.002)	0.011***	(0.002)	0.009***	(0.001)
Child’s Marital Status	-0.023***	(0.022)	-0.090***	(0.020)	0.033***	(0.013)
Age	-0.019***	(0.002)	-0.002	(0.002)	-0.001	(0.001)
Gender	0.244***	(0.017)	0.161***	(0.016)	0.143***	(0.010)
Years of Education	0.007***	(0.002)	0.045***	(0.002)	0.010***	(0.001)
Non-agricultural Population	-0.012	(0.019)	0.117***	(0.021)	-0.009	(0.013)
Urban Area	0.004	(0.006)	0.090***	(0.018)	0.056***	(0.011)
Household Income	0.018***	(0.002)	0.041***	(0.006)	0.029***	(0.004)
House Value	0.070***	(0.023)	0.015***	(0.002)	0.007***	(0.001)
Vehicle Ownership	-0.033	(0.002)	0.047**	(0.021)	0.040***	(0.014)
Educational Expenses	-0.006***	(0.057)	0.012***	(0.002)	-0.002*	(0.001)
Unmarried Ratio	-1.300***	(0.017)	0.35***	(0.054)	0.034	(0.035)
_Cons	1.820***	(0.110)	-1.437***	(0.104)	3.650***	(0.067)
Province Dummy	YES		YES		YES	
R^2^	0.203		0.067		0.037	
Observation	30875		30875		29892	

Note: 1. Interaction = rising housing price*child’s marital status (1 = married, 0 = unmarried). 2. In addition to the *Pols* model, the results obtained from other models are only the original estimated coefficients, not the marginal effects. 3. The value in parentheses is the standard error. 4.***, **, * represent 1%, 5%, and 10% significance levels, respectively.

### Heterogeneity analysis

If rising housing price impairs health of middle-aged and elderly people by strengthening the competitive saving motive, the effect may be heterogeneous among different groups. Given that in China’s marriage market, personal traits (e.g., gender and education) have important impacts on marriage matching [22,23,44,45], we divide child by gender (i.e., male and female) and educational level (i.e., college education or noncollege education) to explore the heterogeneity of the mediation effect of competitive saving motive (Table 6).

**Table 6:** Heterogeneity analysis results.

Dependent Variable	Physical Health		Mental Acuity		Emotional Well-Being	
Model	*probit*		*probit*		*pols*	
Column	(1)	(2)	(3)	(4)	(5)	(6)
Child’s Gender	Male	Female	Male	Female	Male	Female
Rising Housing Price	-0.400***	-0.356***	-0.144***	-0.084***	-0.50***	-0.038**
	(0.017)	(0.022)	(0.016)	(0.020)	(0.010)	(0.013)
Interaction	0.069**	0.058	0.052*	0.027	0.035*	0.020
	(0.026)	(0.033)	(0.023)	(0.030)	(0.015)	(0.019)
R^2^	0.209	0.193	0.068	0.067	0.034	0.043
Observation	18952	11923	18952	11923	18256	11636
Educational Level	College	Non-College	College	Non-College	College	Non-College
Rising Housing Price	-0.498***	-0.370***	-0.104**	-0.132***	-0.038	-0.049***
	(0.048)	(0.018)	(0.044)	(0.016)	(0.025)	(0.010)
Interaction	-0.004	0.061**	-0.072	0.064***	0.04	0.034**
	(0.078)	(0.027)	(0.071)	(0.025)	(0.040)	(0.014)
R^2^	0.277	0.196	0.074	0.061	0.039	0.033
Observation	3666	28412	3666	28412	3620	27469
Edu Expense	YES	YES	YES	YES	YES	YES
Unmarried Gender Ratio	YES	YES	YES	YES	YES	YES
Other Variables	YES	YES	YES	YES	YES	YES
Province Dummy	YES	YES	YES	YES	YES	YES

Notes: 1. Interaction = rising housing price*child’s marriage status. 2. In addition to the *Pols* model, the results obtained from other models are only the original estimated coefficients, not the marginal effects. 3. The value in parentheses is the standard error. 4.***, **, * represent the 1%, 5%, and 10% significance levels, respectively. 5. Due to length limitations, the results of other variables are not reported in this paper but can be obtained from the authors.

In Table 6, the first group divides the gender of the child into male and female. The results show that the interaction between rising housing price and the child’s marital status has a significant positive impact on the physical health, mental acuity and emotional well-being of middle-aged and elderly people with male children, but there is no significant effect for those with female children. The results indicate that the mediation role of competitive saving motive in the impact of rising housing price on the health of middle-aged and elderly people has significant effects in the group with male child, but not in the group with female child. In Table 6, the second group divides the child’s educational level into college and noncollege education. The results show that the interaction between rising housing price and child’s educational level has no significant effect on the physical health, mental acuity and emotional well-being of middle-aged and elderly people with college educated child, while there is a positive significant effect for those noncollege educated child. The results indicate that the mediation effect of competitive saving motive in the impact of rising housing price on the health of middle-aged and elderly people has a significant effect for those with noncollege educated child, but not for those with college educated child. According to the heterogeneity analysis, the effect of the mediation effect of competitive saving motive is heterogeneous with the child’s gender and education level. The results also indirectly indicate that in Chinese traditional marriage culture, the bargaining power of male and female is different; specifically, the burden on the male is significantly higher than that on the female. However, education can offset economic weakness in the marriage match to some extent.

## Conclusions and discussion

Rising housing price and individuals’ health are currently popular topics. In the context of China’s aging population and rising housing price, our study is the first to consider the relationship between rising housing price and the health of middle-aged and elderly people, and divide health into physical health, mental acuity and emotional well-being. First, we adopt the microscopic data from the CFPS national survey in 2010 and 2014, and we choose the middle-aged and elderly people aged 40 and above as our sample. By controlling personal, family, and regional characteristics, the corresponding mixed regression models are used to test the relationship between rising housing price and the health of middle-aged and elderly people. Second, based on the social status seeking theory, we analyze how rising housing price affects the health of middle-aged and elderly people, and use the Casual Step Regression method and the “*Bootstrap*” method to verify the analysis results. Finally, to further contribute to the theory, we analyze whether there are other mediation effects between rising housing price and the health of middle-aged and elderly people, and identify heterogeneous effects, based on the perspective of marriage matching and intergenerational family relationships.

Through a series of theoretical and empirical analyses, we obtain the following robust results: first, rising housing price has a significant negative impact on the health (including physical health, mental acuity, and emotional well-being) of middle-aged and elderly people. Second, the mediation effect of social status seeking motivation between rising housing price and the health of middle-aged and elderly people is significant. It is a mechanism of the main effect, and this mediation effect is partial rather than full. Third, the mediation effect of competitive saving motive between rising housing price and the health of middle-aged and elderly people is significant. It is also a partial mediation effect, which is complementary to the social status seeking motivation. Fourth, the mediation effect of competitive saving motive is significantly heterogeneous. Specifically, the effect is significant for middle-aged and elderly people with male or noncollege educated child, while the effect does not exist for those with female or college educated child.

Based on the conclusions, to improve the current status of health inequality, we propose the following suggestions: first, since rising housing price harms the health of middle-aged and elderly people, the government should try to use macro control measures to curb housing price. Alternatively, “special housing” or housing subsidy policies can be directed based on the situation for some vulnerable groups in China. Second, rising housing price can influence the health of middle-aged and elderly people through social status seeking motivation and competitive saving motive. On the one hand, the government can help residents establish a correct worldview and values, and dilute the sense of competition among residents. On the other hand, the government should standardize and improve certain outdated customs and cultures in the marriage market, and alleviate the burden of marriage matching among residents. Third, the government should promote gender equality and vigorously support higher education. Fourth, intergenerational family relationships are prominent in China, which is a reason why middle-aged and elderly people rely on their children to support them. We also indicate the negative impact of the intergenerational family relationships on the health of middle-aged and elderly people. To alleviate the negative impact, the government should improve and strengthen the social pension security system. Due to the complicated environment, the feasibility of these suggestions should be further investigated.

Our work also has some limitations, which can be addressed in future research. First, the need for buying a house seems to be more pressing for the youth group than for the middle-aged and elderly group. Campbell et al. [53] found that housing price has a statistically significant impact on elderly people, but a weaker impact on young people. We focus on the health effects of rising housing price on middle-aged and elderly people; future studies can explore the impact of rising housing price on the health of youth people and its impact mechanisms. Second, although prior studies rarely consider the measurement of competitive saving motive, limited to research data, we use indirect methods to find the impact of rising housing price on the health of middle-aged and elderly people has a significant “bidding effect”. Compared with the indirect proof method, the direct proof method can better reveal the relationship between various factors, so future research should explore the mediation effect of competitive saving motive by using a direct proof method. Third, there is no denying that the environmental factors have an important impact on individuals’ health; however, due to limited data, we do not control for environmental factors in our model. Future studies should consider environmental factors if possible. Fourth, in the further mechanism analysis, we refer to Yu and Lian [31], and delete the marital status of divorced and widowed samples, so our results do not apply to middle-aged and elderly people with divorced, widowed or no child. Future studies should consider these special groups to enrich existing researches.

## Endnotes

① In the following robustness tests, we use different age ranges for middle-aged and elderly people.

② Income is an important manifestation of individual social status [22]. In view of this, we use individual real income to rank the individual’s actual social status in the local ranking.

③ In Table 2, the results of columns (1) and (2) are the original estimated coefficients, not the marginal effects.

④ Zhao et al [40] believed that the second-order core independent variable estimation coefficient in the causal step regression analysis method is multiplied by the third-order core independent variable estimation coefficient and the mediator estimation coefficient. If the value is positive, it means that there is a complementary mediation; on the contrary, if the value is negative, there is a competitive mediation.

⑤ We refer to the practice of existing literatures and use the average annual growth rate of housing price over the past five years to measure rising housing price.

⑥ In the original CFPS questionnaire, the samples’ options for the question are “1” for “better”, “3” for “no change”, and “5” for “worse”. In this paper, the options representing “no change” and “worse” are assigned as “0” and “-1”, respectively.

⑦ The data comes from the “marriage house” research report released by the well-known Chinese domestic marriage and love dating platform, *Century Jiayuan United Chain Home*.

⑧ The data comes from a sample survey conducted by *Shanghai Daily*.

⑨ The reasons we use the indirect proof method in this study are as follows. First, Wei and Zhang [22] argued that competitive saving motive is the mediation mechanism of gender imbalance and household savings, and used housing price to measure competitive saving motive. Since they do not construct a logical chain of gender imbalance and housing price from the theoretical level, many researchers doubt about this. It indirectly indicates that Wei and Zhang [22] use of housing price to measure competitive saving motive has not been widely recognized by scholars. In addition, we identify whether competitive saving motive plays a mediation effect between rising housing price and the health of middle-aged and elderly people. Based on this purpose, it is not appropriate to use housing price to measure competitive saving motive. Second, although some scholars have used gender imbalance as the research background, and explored the impact of competitive saving motive on consumption, savings and entrepreneurship [22,44,45,51], they merely explained the relationship between competitive saving motive and various dependent variables from the theoretical level, but did not use empirical data to support their research. This suggests that existing research lacks a measure of competitive saving motive.

⑩ Compared with Yu and Lian [31], we lessen the sample restrictions. The main reason is that although a “marriage house” in China is mainly purchased by males, it cannot be ruled out that females are also involved in the purchase of a “marriage house”. This situation has been confirmed by the investigation report jointly published by *Century Jiayuan and Chain Home* (which is one of the Chinese top real estate agencies). Based on the reality of the purchase of a “marriage house” in China, we explore the competitive saving motive by discussing whether the interaction between a child's marital status and housing price is significant. The results of this study are more universal than that of Yu and Lian [31]. In addition, we also draw on the practice of Yu and Lian [31] in the subsequent heterogeneity analysis, and we further identify the heterogeneity of the interaction between a child’s marital status and housing price from the perspective of the child’s gender.

⑪ With reference to Yu and Lian [31], we exclude divorced and widowed samples from the child’s marital status, leaving only unmarried and married samples.

## Supporting information

S1 File (RAR)

S2 File (RAR)

S3 File (RAR)
